# Effects of Intense Pulsed Light on Tissue Vascularity and Wound Healing: A Study with Mouse Island Skin Flap Model

**DOI:** 10.1155/2015/429367

**Published:** 2015-02-03

**Authors:** Trinh Cao Minh, Do Xuan Hai, Pham Thi Ngoc

**Affiliations:** ^1^Practical and Experimental Surgery Department, Vietnam Military Medical University (HVQY), K58, Hadong, Hanoi, Vietnam; ^2^Veterinary Hygiene Department, National Institute of Veterinary Research (NIVR), Truong Chinh, Dong Da, Hanoi, Vietnam

## Abstract

Intense pulsed light (IPL) has been used extensively in aesthetic and cosmetic dermatology. To test whether IPL could change the tissue vascularity and improve wound healing, mice were separated into 4 groups. Mice in Group I were not treated with IPL, whereas, dorsal skins of mice in Groups II, III, and IV were treated with 35 J/cm^2^, 25 J/cm^2^, and 15 J/cm^2^ IPL, respectively. After 2 weeks, dorsal island skin flaps were raised, based on the left deep circumflex iliac vessels as pedicles; then, survival rate was assessed. Flaps in Group IV (treated with lowest dose of IPL) have a survival rate significantly higher than other groups. Counting blood vessels did not demonstrate any significant differences; however, vessel dilation was found in this group. The results show that IPL at the therapeutic doses which are usually applied to humans is harmful to mouse dorsal skin and did not enhance wound healing, whereas, IPL at much lower dose could improve wound healing. The possible mechanism is the dilation of tissue vasculature thanks to the electromagnetic character of IPL. Another mechanism could be the heat-shock protein production.

## 1. Introduction

Wound healing impairment is often encountered in surgery. There are many studies on this phenomenon, but many problems remain unknown and are subject to fierce debates.

To study wound healing, many research models have been developed [1]. In this study, the dorsal island skin flap model in the mouse has been used. This model has been pioneered by the author in the previous studies [2, 3]. The advantages and disadvantages of this model for wound healing study will be discussed along with relevant background information to help guide decision-making.

A wound is associated with the interruption of the blood supply (ischemia). The healing of a wound, besides many other mechanisms, is involved with the process of the resupply of blood (reperfusion). It is the decisive factor for healing.

Recently, it was found that electromagnetic radiation at low dose could improve the tissue perfusion of the irradiated tissue [4]. It has been known for long time that light can greatly affect the health. Light also has the character of the electromagnetic radiation. Therefore, it is reasonable to suggest that light might be able to affect the wound healing process.

Intense pulsed light (IPL) systems are high intensity pulsed sources that emit polychromatic light in a broad wavelength spectrum. The emitted broadband light is covering the spectrum from ultraviolet (UV) to infrared (IR). However, the most common systems emit radiations between 400 and 1,200 nm. When using IPL for cutaneous treatment, the reaction mechanism of the IPL sources is based on the principle of selective photothermolysis [5].

IPL devices are being used for a diverse range of treatments like photorejuvenation, for acne or cellulite and inflamed, and hypertrophic scars or keloids. It is safe and common that the novel, low-fluence, home-use IPL devices for hair removal have entered the consumer market [6].

IPL can make skin “stronger and younger,” IPL has character of electromagnetic radiation, and IPL can induce heat-shock protein production [7]. Can IPL improve the wound healing in the skin? This study was carried out to check whether IPL could change the tissue vascularity and improve wound healing.

## 2. Materials and Methods

### 2.1. Animals

Adult, male, and ICR mice (30–40 gr) were acclimatized to their holding facility for at least 5 days before experimental manipulation. The animals were treated according to the Animal Regulations of the Vietnam Military Medical University published in 1989.

### 2.2. Experiment Protocol

#### 2.2.1. Allocation of Animals

160 mice were used and separated into 4 groups. Group I served as control. Two weeks before operation, dorsal skin of mice in Group II was treated with 35 J/cm^2^ IPL, Group III with 25 J/cm^2^, and Group IV with 15 J/cm^2^.

40 mice (10 mice per group) were sacrificed for histological study; other 120 mice (30 mice per group) were used to create skin flaps.

#### 2.2.2. Intense Pulsed Light (IPL) Treatment Parameters

Effect of IPL was investigated in 120 mice. Animals were randomly assigned to one of the 3 groups (Group II, Group III, and Group IV), 40 mice per group. Before receiving IPL treatment, mice were anesthetized with pentobarbital sodium (1 mg/mL), diluted 5 times with saline, and injected intraperitoneally (0.001 mg/gr). Dorsal hair was removed with electric clippers and depilatory cream. Dorsal skins were treated with a light emission apparatus of the Lumenis One IPL system with a Universal IPL handpiece in a single session. A 515 nm cut-off filter, triple pulse mode with pulse length of 5 ms and delay of 30 ms, was used for all treatment. However, mice in Group II, Group III, and Group IV were treated with different fluences of 35 J/cm^2^, 25 J/cm^2^, and 15 J/cm^2^, respectively. The skin area was treated 3 times with 5-minute interval. During the procedure, chilled, colorless gel was used to aid in delivering the light uniformly onto the skin surface and to reduce the thermal effect of IPL. After IPL treatment, mice were observed daily for 2 weeks until the time of the skin flaps elevation.

#### 2.2.3. Surgical Procedure and Flap Survival Assessment

120 mice were used to create skin flaps. Among them, in Group I (control group), skin flaps were raised without IPL treatment, whereas in Group II, Group III, and Group IV, skin flaps were raised at the time of two weeks after IPL treatment.

**Figure 1:**
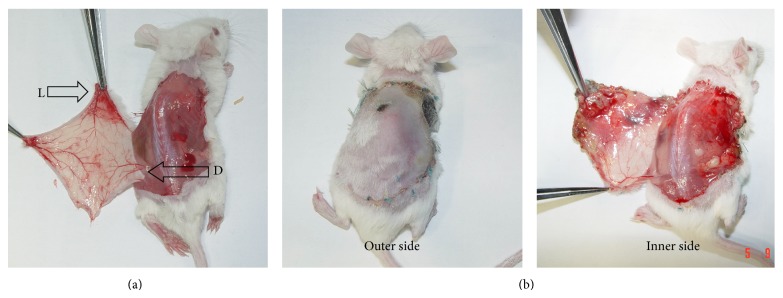
Dorsal island skin flap. (a) (D) the left deep circumflex iliac vessels as pedicle and (L) the left lateral thoracic vessels. (b) Flap survival and necrosis determination.

Design and surgical technique for the elevation of the dorsal island skin flap in the mouse have been described in the author's previous studies [2, 3]. In brief, mice were anesthetized with pentobarbital sodium (1 mg/mL), diluted 5 times with saline, injected intraperitoneally (0.001 mg/gm), and supplemented as necessary. Dorsal hair was removed with electric clippers and depilatory cream. The entire dorsal island skin flap was elevated based on the left deep circumflex iliac vessels as pedicles (Figure 1(a)). Both halves of this flap consisted of 2 adjacent vascular territories: the deep circumflex iliac artery and the lateral thoracic artery territories. If the vascular pattern deviated from the abovementioned pattern, the mouse was excluded from the study.

The flap was elevated from the cranial side to the caudal side. An operating microscope (Zeiss, Germany) was used for dissecting out the vascular pedicles. Flaps were then resutured in position using interrupted 6/0 nylon sutures.

Flap survival and necrosis were determined at the 6th postoperative day by the method described previously [8]. In brief, both outer and inner sides of the flap were checked. Skin necrosis was defined grossly by typical signs of tissue injury on the outer side: black color, dehydration, and eschar formation, and on the inner side, no vasculature (Figure 1(b)).

The total area of the flaps and the survival area were traced on clear acetate sheets and scanned as digital images. The digital images were analyzed using Adobe Photoshop CS3 Extended software (Adobe Systems, Inc., San Jose, CA) to calculate the percentage of the survival area. Measurements were performed twice, and mean values were used for statistical analysis. The survival rate was expressed as the percentage of the surviving area to the total skin flap area.

### 2.3. Histological Study

40 mice of 4 groups (10 mice per group) were sacrificed for histological study. The skin samples were taken as 7 × 7 mm biopsies from the center of dorsal skin of mice in Group I (control group). The same was done with mice in Group II, Group III, and Group IV, but at the time of 2 weeks after IPL treatment.

Skin biopsies were stained with hematoxylin and eosin. Blood vessels were counted in histological sections by the method described elsewhere [9]. Blood vessels were evaluated in light microscopy at 200x magnification. Number of vessel was counted in five fields of each section. Results are presented as mean number of vessel per field ± SD. At the same time, vessel diameter was measured with the use of an eyepiece micrometer (reticle) and a stage micrometer. Results are presented as mean diameter of vessels per field ± SD.

### 2.4. Statistical Analysis

All data are presented as mean ± SD in the text and figures. The Student *t*-test was used to compare two means. Statistical significance was set at a *P* < 0.05 level.

## 3. Results

Table 1 shows the allocation of mice in the different groups of the study, as well as the number of excluded and participating animals.

Several vascular patterns of mouse dorsal skin have been recorded in this study. In the most frequently observed pattern, 114/120 (95%), the mouse dorsal skin was supplied by the two principal cutaneous perforators, which arose bilaterally from the deep circumflex iliac artery and the lateral thoracic artery.

**Figure 2:**
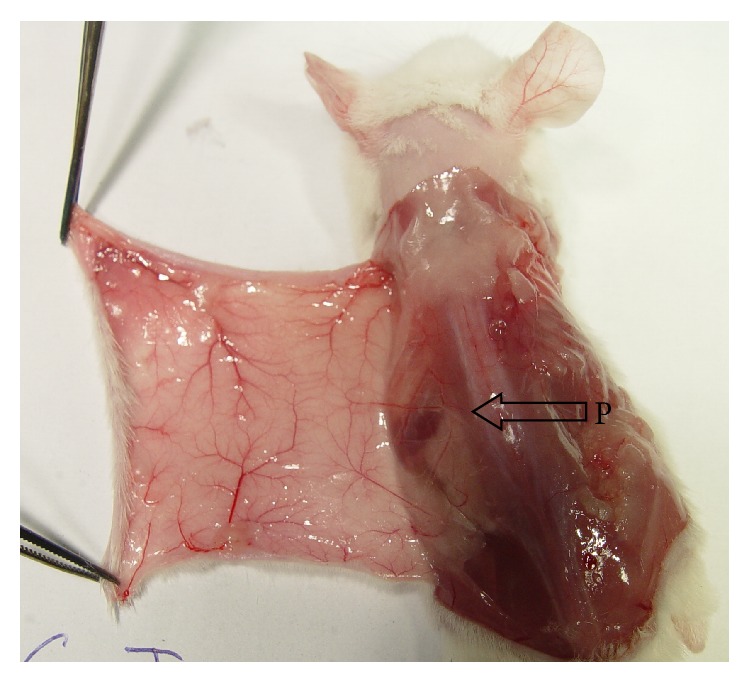
An example of abnormal vasculature. (P) a branch of the posterior intercostals arterial system.

6/120 (5%) mice have abnormal vasculature.  Figure 2 shows an example of abnormal vasculature. In this case, the middle portion of dorsal skin in the left was supplied by a branch of the posterior intercostals arterial system. Such cases will be excluded from the study.

**Figure 3:**
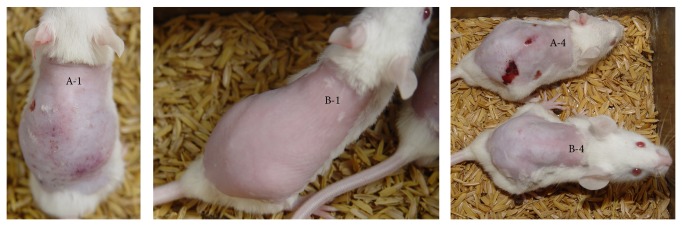
Injury caused by IPL. (A-1) mouse got 35 J/cm^2^ IPL fluences, one day after treatment, (A-4) mouse got 35 J/cm^2^ IPL fluences, 4 days after treatment, (B-1) mouse got 25 J/cm^2^ IPL fluences, one day after treatment, and (B-4) mouse got 25 J/cm^2^ IPL fluences, 4 days after treatment.

In 120 mice of IPL-treated groups, IPL with fluences of 35 J/cm^2^ (Group II) has caused injury of the dorsal skin in 12/40 (30%) mice, whereas IPL with fluences of 25 J/cm^2^ and 15 J/cm^2^ has not caused any injury. The injury cannot be recognized immediately after IPL treatment. But it is able to be foreseen one day after IPL treatment and clearly seen four days after IPL treatment (Figure 3).

Table 2 shows the average survival rates of skin flaps from different groups of the study. Effect of IPL on wound healing was investigated based on the survival rates of skin flaps from different groups of the study.

**Figure 4:**
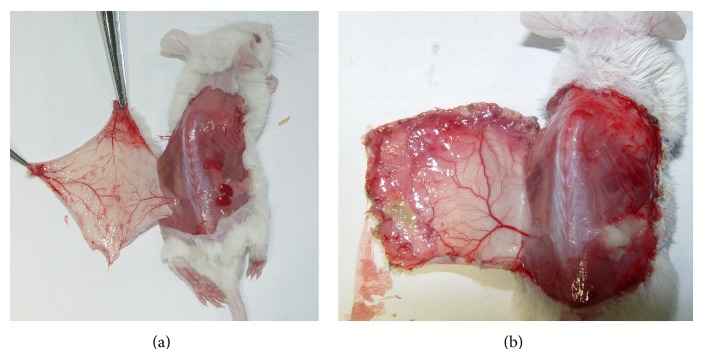
Changing of vasculature and neovascularization. (a) Vasculature at the time of flap elevation and (b) vasculature at the 6th postoperative day: changing and neovascularization could be clearly seen.

In Group I (control group), skin flaps were raised without IPL treatment. At the 6th postoperative day, both outer and inner sides of the flap were checked. Change of vasculature was clearly seen at this day (Figure 4). Skin flaps average survival rate was 37.8 ± 10.6 (mean ± SD).

In Groups II and III, skin flaps survival rates are 35.2 ± 8.9 and 48.7 ± 12.5 respectively. There is no significant difference between these groups. Only in Group IV (mice treated with IPL fluences of 15 J/cm^2^, lowest dose), skin flaps survival rate is significantly higher than other groups: 69.7 ± 15.9 (*P* < 0.05).

Table 3 shows the number of vessels and vessels diameter from different groups of the histological study.

Histological study did not find any significant differences in vessels number of any groups, with or without IPL treatment. However, vessels dilation was found in Group IV; the group was treated with lowest dose of IPL (15 J/cm^2^).

**Figure 5:**
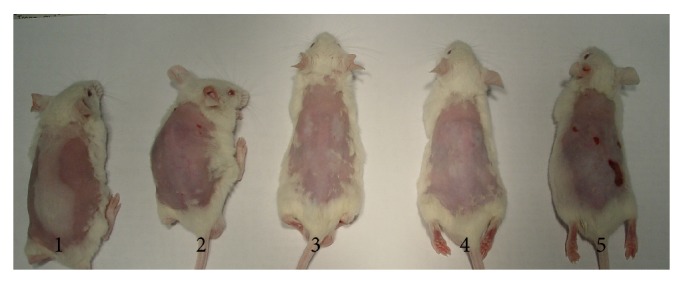
Hair reduction effect of IPL on mice.

Hair regrowth is not primary consideration of this study. However, in 120 mice treated with IPL, it was recognized that hair regrowth did not follow any defined rule. Mice get the same treatment, but hair regrowth of each mouse is quite different (Figure 5).

## 4. Discussion

### 4.1. Mouse Island Skin Flap Model

Wound healing is a very complicated process. To study the basic mechanism of healing, to develop strategies for clinical treatment, and to evaluate the product safety and efficacy, a suitable animal wound model is indispensable [10]. Many different models have been devised [11]. An ideal wound model should reflect the wound pathogenesis and illustrate the clinical situation [1]. However, no ideal wound model could reflect all aspects of wound healing process [1, 11].

Blood supply to a wounded tissue is the prerequisite for normal tissue regeneration. Up to date, vascular change in the healing process of skin wounds is not fully understood. To study vascular change, installing a vital chamber to mouse dorsal skin is a good option, but it requires much expensive equipment [12].

In this study, our model can reflect one important factor of wound healing. It is the vascular change in the healing process of the ischemic skin flap. By counting the number of vessels in biopsies samples and looking to the vasculature from inner side of mouse skin (Figure 4), researchers can assess the neovascularization process. In this model, the neovascularization process was permitted to develop in the parallel way (from the proximal part to the distal part of the skin flap) instead of perpendicular way from wound bed as in other models.

Mice were used in this study. Skin flap models on dogs [13] and on rats [14] have been described. Flaps on mice offer clear advantage. Mice are inexpensive and easy to keep and gene modified mice are also available.

In comparison with the vital chamber model [12], this model is simple, cheap, and, therefore, suitable for less-equipped labs in developing countries. Obviously, continuous observation of vasculature is impossible. It is the disadvantage of this model.

### 4.2. Effect of IPL on Vascularity and Wound Healing

The novelty of this study is the effect of IPL on wound healing. We have not found any other reports about this particular approach to IPL use.

The results showed that the flap survival was significantly improved in the mice that had been treated with IPL fluences of 15 J/cm^2^—the lowest dose. Interestingly, 15 J/cm^2^ is a very low fluence in comparison with normal therapeutic fluences used on humans. We are not aware of any clinical studies using such low fluences. A higher dose (fluences of 35 J/cm^2^ and 25 J/cm^2^) did not produce any improvement on wound healing. Actually, fluences of 35 J/cm^2^ have caused injury of the dorsal skin in 12/40 (30%) mice of Group II. By these results, one could say that the IPL effect is dose dependent. IPL can significantly improve the wound healing but only at low energy. The effect will be lost at high energy.

What is the mechanism of this phenomenon? Histological study has not found any tissue destruction after treatment with IPL fluences of 15 J/cm^2^ (the effective dose). So, the possible mechanism must be different from tissue selective destruction mechanism which is generally used in human clinical situations [5].

Angiogenesis cannot be assumed to be the cause of wound healing improvement in this case. Vessel counting did not find any significant differences in vessel number of any groups, with or without IPL treatment (Table 3).

However, vessel dilation was found in Group IV; the group was treated with lowest dose of IPL and wound healing improved (Table 3). IPL produces heat in tissue. It is well known that, in the increased temperature, the cutaneous vessel becomes dilated as a way to expel the heat but this phenomenon will not last for long. Therefore, vessel dilation found 2 weeks after IPL treatment cannot be assumed to be caused by the heating effect of IPL. Possibly, it is due to the electromagnetic character of IPL. It was reported that the electromagnetic radiation at low dose could improve the tissue perfusion of the irradiated tissue [4].

Another possible explanation for wound healing enhancement of IPL treatment is the production of heat-shock protein after IPL treatment [7]. Then, heat-shock protein could protect tissue from ischemia injury and improve the healing [3].

It is not the main purpose of this study, but interestingly, it was found that hair reduction effect of IPL is not uniform from mouse to mouse (Figure 5). It resembles the clinical situation in humans. With the same IPL treatment, response of patients is quite different.

Results of this study cannot be supposed to be equally effective in human because of species differences. Mouse skin is much thinner than human skin. Even though IPL fluences of 15 J/cm^2^ (which effectively improves wound healing) are just equal to the energy of home-use IPL devices that are freely available in the consumer market, such devices do not present any hazards according to currently available international standards [6].

## 5. Conclusion

In this study, we have shown that the dorsal island skin flap in the mouse is a suitable model for wound healing research purpose. This model is reliable and can offer some advantages for the study of wound healing where ischemia injury plays crucial role.

For the first time, the study has demonstrated that low-energy IPL augments wound healing. However, the exact mechanism is not clear at this stage of the study. Obviously, time and further research are needed to elucidate the true mechanism.

Therefore, we suggest using low-energy IPL as an adjuvant therapy to improve wound healing in humans because low-energy IPL devices are freely available for home use and do not present any hazard.

## Figures and Tables

**Table 1:** Number of used and excluded mice in the study.

Groups and IPL treatment fluences	Number of mice in each group	Number of mice used for histological Study	Number of mice used to create skin flaps and flap survival assessment			
			Excluded and reasons			Used for study
			Abnormal vasculature	Pedicle not patent	Died	
Group I (control)	40	10	1	1	1	27

Group II (35 J/cm^2^)	40	10	1		1	28

Group III (25 J/cm^2^)	40	10	1			29

Group IV (15 J/cm^2^)	40	10	3		1	26

Total	160	40	6	1	3	110

**Table 2:** Survival rates of skin flaps from different groups of the study.

Groups and IPL treatment fluences	Number of mice in each group	Number of mice used for flap survival assessment	Survival rate of skin flaps (%)
Group I (control)	40	27	37.8 ± 10.6 (mean ± SD)

Group II (35 J/cm^2^)	40	28	35.2 ± 8.9 (mean ± SD)

Group III (25 J/cm^2^)	40	29	48.7 ± 12.5 (mean ± SD)

Group IV (15 J/cm^2^)	40	26	69.7 ± 15.9 (mean ± SD) **P** ** < 0.05**

Total	160	110	

**Table 3:** Number of vessels and vessels diameter from different groups of the histological study.

Groups and IPL treatment fluences	Number of mice used for histological Study	Number of vessels per field	Diameter of vessels per field (*µ*m)
Group I (control)	10	25.4 ± 6.2 (mean ± SD)	8.8 ± 3.6 (mean ± SD)

Group II (35 J/cm^2^)	10	26.7 ± 7.1 (mean ± SD)	7.9 ± 4.2 (mean ± SD)

Group III (25 J/cm^2^)	10	23.2 ± 8.3 (mean ± SD)	8.7 ± 2.5 (mean ± SD)

Group IV (15 J/cm^2^)	10	25.7 ± 5.4 (mean ± SD)	14.7 ± 2.1 (mean ± SD) **P** ** < 0.05**

Total	40		
